# Water Wave Solutions of the Coupled System Zakharov-Kuznetsov and Generalized Coupled KdV Equations

**DOI:** 10.1155/2014/724759

**Published:** 2014-10-12

**Authors:** A. R. Seadawy, K. El-Rashidy

**Affiliations:** ^1^Mathematics Department, Faculty of Science, Taibah University, Al-Ula 41921-259, Saudi Arabia; ^2^Mathematics Department, Faculty of Science, Beni-Suef University, Beni-Suef, Egypt; ^3^Mathematics Department, College of Arts and Science, Taif University, Ranyah, Saudi Arabia

## Abstract

An analytic study was conducted on coupled partial differential equations. We formally derived new solitary wave solutions of generalized coupled system of Zakharov-Kuznetsov (ZK) and KdV equations by using modified extended tanh method. The traveling wave solutions for each generalized coupled system of ZK and KdV equations are shown in form of periodic, dark, and bright solitary wave solutions. The structures of the obtained solutions are distinct and stable.

## 1. Introduction

Many nonlinear evolution equations are playing important role in the analysis of some phenomena. In the study of equations modeling wave phenomena, one of the fundamental objects is the traveling wave solution. Traveling wave solution expressions are in explicit or implicit forms. These types of waves will not change their shapes during propagation. The particular interests are three types of traveling waves: the solitary waves, which are localized traveling waves, asymptotically zero at large distances, the periodic waves, and the kink waves, which rise or descend from one asymptotic state to another. A unified method, called the extended mapping method, is developed to obtain exact traveling wave solutions for a large variety of nonlinear partial differential equations [1, 2]. By means of this method, the solitary wave, the periodic wave, and the kink wave solutions can be obtained simultaneously. In order to describe complex phenomena in various fields of science, some important nonlinear evolution equations have been established, such as Kadomtsev Petviashvili (KP) equation, Korteweg-de Vries (KdV) equation, and Zakharov-Kuznetsov (ZK) equation [3]. The KdV equation is a model to describe and identify mechanisms for atmospheric blocking. The ZK equation governs the behavior of weakly nonlinear ion-acoustic waves in plasma comprising cold ions and hot isothermal electrons in the presence of a uniform magnetic field [4, 5]. Moreover, ZK equation supports stable solitary waves, which makes ZK equation a very attractive model equation for the study of vortices in geophysical flows [5, 6].

The ZK equation was first derived for describing weakly nonlinear ion-acoustic waves in strongly magnetized lossless plasma in two dimensions [4]. Wazwaz [7] used extended tanh method for analytic treatment of the ZK equation, the modified ZK equation, and the generalized forms of these equations. Huang [8] applied the polynomial expansion method to solve the coupled ZK equations. Zhao et al. [9] obtained numbers of solitary waves, periodic waves, and kink waves using the theory of bifurcations of dynamical systems for the modified ZK equation. Inc [10] solved nonlinear dispersive ZK equations using the Adomian decomposition method, and Biazar et al. [11] applied the homotopy perturbation method to solve the ZK equations. In [12], the approximate analytical solution of a Zakharov-Kuznetsov ZK(*m*, *n*, *k*) equation with the help of the differential transform method (DTM) is presented. The DTM method is a powerful and efficient technique for finding solutions of nonlinear equations without the need of a linearization process. The DTM is an analytical method based on a Taylor expansion. This method constructs an analytical solution in the form of a polynomial [13–15]. The application of DTM is successfully extended to obtain analytical approximate solutions to various linear and nonlinear problems [16, 17].

The coupled KdV system, since Hirota and Satsuma presented the first coupled KdV system [18], its properties have been researched amply [19–21]. After that, a series of important coupled KdV models are constructed [22]. Some kinds of general coupled KdV equations gain real application in some fields such as in shallow stratified liquid [23–25], atmospheric dynamical system [26], and two-component Bose-Einstein condensates [27]. Lou et al. [28] obtained exact solutions of a coupled KdV system with a formally variable separation approach and derived a coupled variable coefficient mKdV equation from a two-layer fluid system [29]. Hu et al. [30] discovered nonsingular positon and complexiton solutions for a special coupled KdV system by means of the iterative Darboux transformation. The research about solution, structure, interaction, and other properties of soliton abstracts much more attention and many meaningful results are obtained successfully [31–35].

This paper is organized as follows. An introduction in Section 1. In Section 2, the formulation of stability analysis solutions. In Section 3, we found the exact soliton solutions for the coupled system of ZK equations. The travelling wave solutions of the generalized system of KdV equations are obtained in Section 4. Finally, the paper end with a conclusion in Section 5.

## 2. Stability of Solutions

Hamiltonian system for which the momentum is given by

(1) $$\begin{array}{c} M=\frac{1}{2}\int \overset{\infty }{\underset{-\infty }{\int }}U_{ij}^{2}\left(t,x\right)dt\;dx,\;i=1,2,\;\;j=1,2,3, \end{array}$$

where *U*_1_ = *u*(*x*, *t*) and *U*_2_ = *v*(*x*, *t*). The sufficient condition for discussing the stability of solution ∂*M*/∂*k* > 0.

## 3. The Generalized Coupled ZK Equations

The general forms of the coupled ZK equations [8] are

(2) $$\begin{array}{c} u_{t}+u_{xxx}+u_{yyx}-6uu_{x}-v_{x}=0,\\ v_{t}+\delta v_{xxx}+\lambda v_{yyx}+\eta v_{x}-6\mu vv_{x}-\alpha u_{x}=0. \end{array}$$

The coupled ZK equations are the model describing two interacting weakly nonlinear waves in anisotropic background stratified followed flows. Here, *x* and *y* are the propagation and transverse coordinates, *η* is a group velocity shift between the coupled models, *δ* and *λ* are the relative longitudinal and transverse dispersion coefficient, and *μ* and *α* are the relative nonlinear and coupled coefficients. In case [*u*_*y*_ = *v*_*y*_ = 0], this system reduces to the set of coupled KdV equations. To look for the traveling wave solutions of coupled ZK equation (2). Consider the traveling wave solutions:


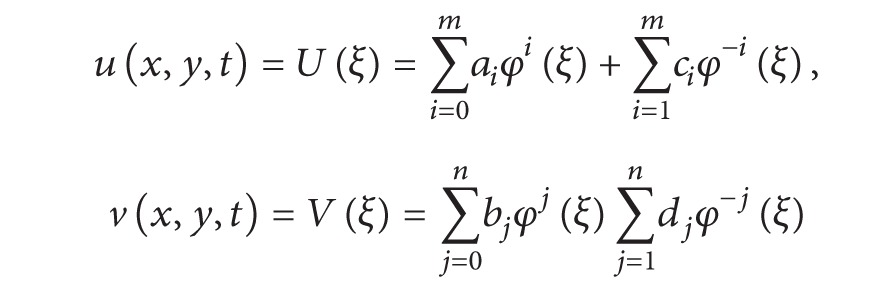

(3)



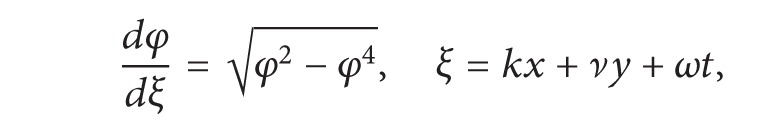

(4)

where *a*_*i*_, *c*_*i*_, *b*_*j*_, *d*_*j*_, *ν*, *k*, and *ω* are arbitrary constants and *m* and *n* are positive integers, in most cases, that will be determined. The parameters *m* and *n* are usually obtained by balancing the linear terms of the highest order in the resulting equation with the highest order nonlinear terms. Substituting (3) into (2), (2) becomes

(5) $$\begin{array}{c} \left(k^{3}+\nu^{3}\right)U^{\prime \prime }+\left(\omega -3kU\right)U-kV=0,\\ \left(\delta k^{3}+\lambda k\nu^{2}\right)V^{\prime \prime }+\left(\omega +k\eta -6k\mu V\right)V-\alpha kV=0. \end{array}$$

We suppose that the solution of (5) is in the following form:

(6) $$\begin{array}{c} U\left(\xi \right)=a_{0}+a_{1}\varphi +c_{1}\varphi^{-1}+a_{2}\varphi^{2}+c_{2}\varphi^{-2},\\ V\left(\xi \right)=b_{0}+b_{1}\varphi +d_{1}\varphi^{-1}+b_{3}\varphi^{2}+d_{2}\varphi^{-2}. \end{array}$$

Substituting (6) into (5) yields a set of algebraic equations for *a*_0_, *a*_1_, *a*_2_, *c*_1_, *c*_2_, *b*_0_, *b*_1_, *b*_2_, *d*_1_, *d*_2_, *α*, *δ*, *λ*, *ν*, *ω*, *k*, *μ*, and *η*. We have two cases for these equations that are found as follows.

*Case 1.* In the solution of the system of (5), we can find

(7) $$\begin{array}{c} a_{0}=-\frac{\alpha +\eta +\omega }{6\mu },\;\;a_{1}=-1,\;\;a_{2}=c_{2}=0,\\ c_{1}=\frac{3k\left(\alpha +2\mu \right)}{\alpha \left(3k+\omega \right)}, \end{array}$$

(8) $$\begin{array}{c} b_{0}=\frac{\alpha +\eta +\omega }{6\mu },\;\;b_{1}=-1,\;\;b_{2}=d_{2}=0,\\ d_{1}=\frac{3\left(k\alpha +8k\mu +2\mu \omega \right)}{\alpha \left(3k+\omega \right)}. \end{array}$$

Substituting (7) and (8) into (6) with *ν* = −*k* and *δ* = −*λ*, we have obtained the following solutions of (2):

(9) u(x,y,t)=−α+η+ω6μ−sech(kx+νy+ωt) +3k(α+2μ)α(3k+ω)sech2(kx+νy+ωt),

(10) v(x,y,t)=α+η+ω6μ−sech(kx+νy+ωt) +3(kα+8kμ+2μω)α(3k+ω)sech2(kx+νy+ωt).

*Case 2.* The solutions of the system of (5) can be found as follows:

(11) $$\begin{array}{c} a_{0}=\frac{2\alpha -\eta +\omega }{3\mu },\;\;a_{1}=2,\;\;a_{2}=c_{2}=0,\\ c_{3}=\frac{12k\left(\alpha +2\mu \right)}{\alpha \omega }, \end{array}$$

(12) $$\begin{array}{c} b_{0}=\frac{-2\alpha +\eta +\omega }{6\mu },\;\;b_{1}=2,\;\;b_{2}=d_{2}=0,\\ d_{1}=\frac{12\left(k\alpha +2k\mu +2\mu \omega \right)}{\alpha \omega }. \end{array}$$

Substituting (11) and (12) into (6), we obtained the following solutions of (2):

(13) u(x,y,t)=2α−η+ω3μ+2 sech(kx+νy+ωt) +12k(α+2μ)αωsech2(kx+νy+ωt),

(14) v(x,y,t)=−2α+η+ω6μ+2 sech(kx+νy+ωt) +12(kα+2kμ+2μω)αωsech2(kx+νy+ωt).

**Figure 1:**
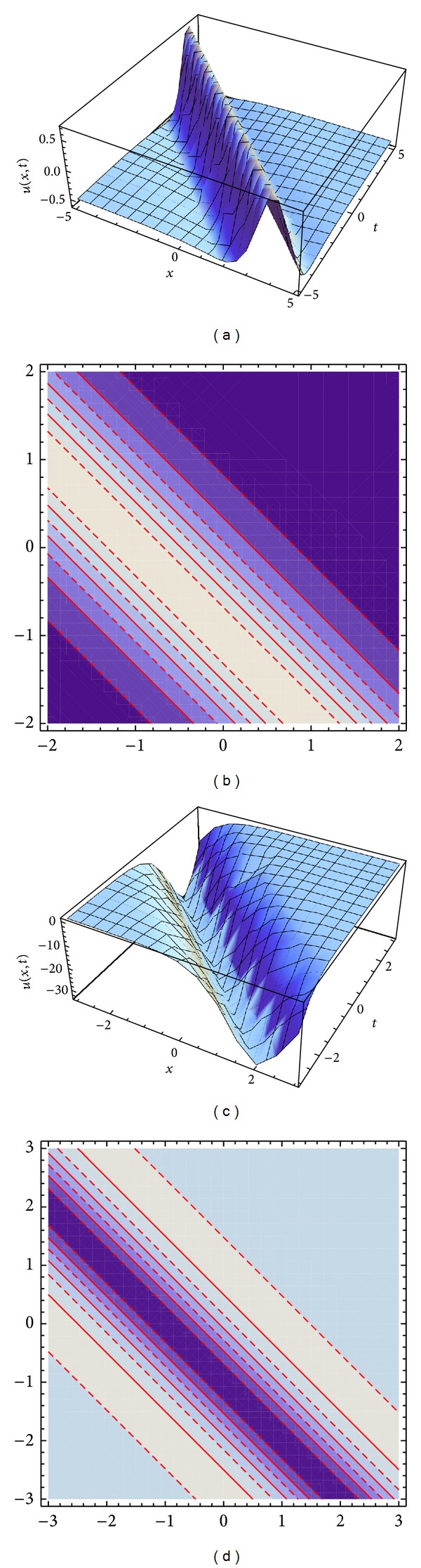
Travelling waves solutions (9) and (10) with various different shapes are plotted: bright solitary waves in (a) and contour plot in (b). Travelling waves solutions (13) and (14) with various different shapes are plotted: dark solitary waves in (c) and contour plot in (d).

Figures 1(a) and 1(c) represent the evolution of the bright and dark solitary wave solutions (9) and (13) of the generalized coupled system ZK equation (2), with *α* = *η* = *ω* = *μ* = *ν* = 1, and *k* = −1. The solitary wave solutions (8)-(9) are stable in the intervals [−5,5] and [−3,3]. A contour plots Figures 1(b) and 1(d) are a collection of level curves drawn on the same set of axes. The Mathematica command ContourPlot draws contour plots of functions of two variables. The contours join points on the surface having the same height. The default is to have contours corresponding to a sequence of equally spaced values of the function.

## 4. The Generalized Coupled KdV Equation

Consider the following generalized coupled KdV equations:

(15) $$\begin{array}{c} u_{t}+\alpha_{1}vu_{x}+{\left(\alpha_{2}v^{2}+\alpha_{3}uv+\alpha_{4}u_{xx}+\alpha_{5}u^{2}\right)}_{x}=0,\\ v_{t}+\delta_{1}vu_{x}+{\left(\delta_{2}u^{2}+\delta_{3}uv+\delta_{4}v_{xx}+\delta_{5}v^{2}\right)}_{x}=0, \end{array}$$

where *α*_*i*_, *δ*_*i*_ (*i* = 1,2, 3,4, 5) are arbitrary constants. This system is derived from two-layer fluids, whose integrability and existence of the solitarty wave solutions for this system have been discussed by Lou et al. [26]. Let us consider the traveling wave solutions *u*(*x*, *t*) = *U*(*ξ*), *v*(*x*, *t*) = *V*(*ξ*), and *ξ* = *x* − *ct*, and then (15) becomes

(16) $$\begin{array}{c} -cU^{\prime }+\alpha_{1}VU^{\prime }+{\left(\alpha_{2}V^{2}+\alpha_{3}UV+\alpha_{4}U^{\prime \prime }+\alpha_{5}U^{2}\right)}^{\prime }=0,\\ -cV^{\prime }+\delta_{1}VU^{\prime }+{\left(\delta_{2}U^{2}+\delta_{3}UV+\delta_{4}V^{\prime \prime }+\delta_{5}V^{2}\right)}^{\prime }=0. \end{array}$$

We assume that *α*_1_ = *δ*_1_ = 0 and *α*_4_ = *δ*_4_ = 1, so (16) becomes

(17) $$\begin{array}{c} -cU+\left(\alpha_{2}V^{2}+\alpha_{3}UV+U^{\prime \prime }+\alpha_{5}U^{2}\right)=0,\\ -cV+\left(\delta_{2}U^{2}+\delta_{3}UV+V^{\prime \prime }+\delta_{5}V^{2}\right)=0. \end{array}$$

Balancing the nonlinear term *UV* and the highest order derivative *U*′′ gives *m* = 2. We suppose that the solution of (17) is in the forms

(18) $$\begin{array}{c} U\left(\xi \right)=a_{0}+a_{1}\varphi +a_{2}\varphi^{-1}+a_{3}\varphi^{2}+a_{4}\varphi^{-2},\\ V\left(\xi \right)=b_{0}+b_{1}\varphi +b_{2}\varphi^{-1}+b_{3}\varphi^{2}+b_{4}\varphi^{-2}. \end{array}$$

Substituting (18) into (17) yields a set of algebraic equations for *a*_0_, *a*_1_, *a*_2_, *a*_3_, *a*_4_, *b*_0_, *b*_1_, *b*_2_, *b*_3_, and *b*_4_. We have two cases for these equations that are found as follows.

*Case 1.* In the solution of the system of (17), we can find

(19) $$\begin{array}{c} a_{0}=1,\;\;a_{1}=\pm \sqrt{\frac{85}{6}},\;\;a_{2}=\mp 9\sqrt{\frac{3}{170}},\\ a_{3}=1,\;\;a_{4}=\frac{343}{340},\\ b_{0}=0,\;\;b_{1}=\pm \sqrt{510},\;\;b_{2}=0,\\ b_{3}=\frac{97}{6},\;\;b_{4}=\frac{243}{85}, \end{array}$$

with *α*_2_ = 1, *α*_5_ = *δ*_5_ = 6, *α*_3_ = −6, *δ*_2_ = 36, *δ*_3_ = −36, and *c* = 4.

Substituting (19) into (18), we have obtained the following solutions of (15):

(20) u(x,t)=1±856sech(x−ct)∓93170cosh⁡(x−ct) + sech2(x−ct)+343340cosh⁡2(x−ct),v(x,t)=±510 sech(x−ct)+976sech2(x−ct) +24385cosh⁡2(x−ct).

*Case 2.* In the solution of the system of (12), we find

(21) $$\begin{array}{c} a_{0}=1,\;\;a_{1}=\frac{1}{45}\left(\pm 4\sqrt{381}\pm \sqrt{10146}\right),\\ a_{2}=\pm \sqrt{\frac{127}{75}},\;\;a_{3}=1,\;\;a_{4}=\frac{1}{2},\\ b_{0}=0,\;\;b_{1}=\frac{2}{15}\left(\pm 4\sqrt{381}\pm \sqrt{10146}\right),\\ b_{2}=0,\;\;b_{3}=16,\;\;b_{4}=2. \end{array}$$

Substituting (19) into (18), we have obtained the following solutions of (15):

(22) u(x,t)=1+145(±4381±10146)sech(x−ct)±12775cosh⁡(x−ct)+sech2(x−ct)+12cosh⁡2(x−ct),v(x,t)=215(±4381±10146)sech(x−ct)+16 sech2(x−ct)+2cosh⁡2(x−ct).

**Figure 2:**
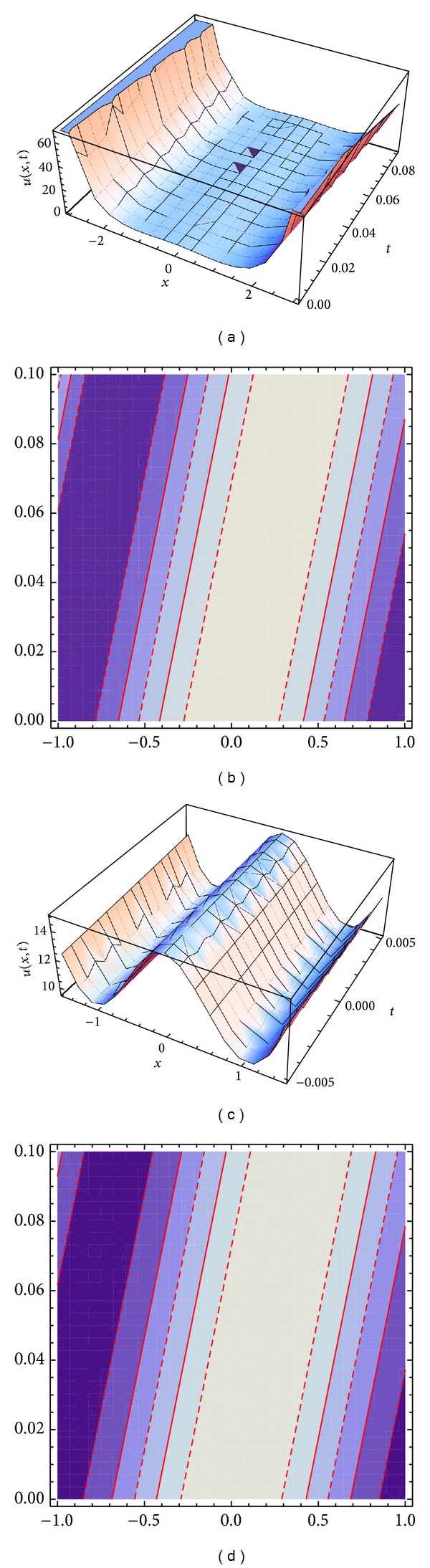
Travelling waves solution (20) with various different shapes are plotted: dark solitary waves in (a) and contour plot in (b). Travelling waves solution (22) with various different shapes are plotted: periodic solitary waves in (d) and contour plot in (c).

Figures 2(a) and 2(b) represent the evolution of the dark and periodic solitary wave solutions (20) and (22) of the generalized coupled system KdV equation (15), with *α*_2_ = 1, *α*_5_ = *δ*_5_ = 6, *α*_3_ = −6, *δ*_2_ = 36, *δ*_3_ = −36, and *c* = 4. The solitary wave solutions (20) and (22) are in the intervals [0,1] and [−2,2].

## 5. Conclusion

The basic goal of this work has been the study of a generalized ZK equations, which is important in mathematics and physics. The explicit solutions of GZK equations, KdV system equations, and KdV equation are obtained. These exact solutions might provide a useful help for physicists to study more complicated physical phenomena. All soliton solutions are exact and stable and have applications in physics.
